# Telomere Length and COVID-19 Severity: A Comparative Cross-Sectional Study Across the Clinical Spectrum

**DOI:** 10.3390/healthcare13202656

**Published:** 2025-10-21

**Authors:** Flora Bacopoulou, Anastasios Tentolouris, Eleni Koniari, Dimitrios Kalogirou, Dimitrios Basoulis, Ioanna Eleftheriadou, Pinelopi Grigoropoulou, Vasiliki Efthymiou, Konstantina K. Georgoulia, Ioanna A. Anastasiou, Stavroula Papadodima, George Chrousos, Nikolaos Tentolouris

**Affiliations:** 1 Center for Adolescent Medicine and UNESCO Chair in Adolescent Health Care, First Department of Pediatrics, School of Medicine, National and Kapodistrian University of Athens, Aghia Sophia Children’s Hospital, 11527 Athens, Greece; bacopouf@hotmail.com; 2 First Department of Propaedeutic Internal Medicine and Diabetes Center, School of Medicine, National and Kapodistrian University of Athens, Laiko General Hospital, 11527 Athens, Greece; antentol@med.uoa.gr (A.T.); joeleftheriadou@yahoo.com (I.E.); anastasiouiwanna@gmail.com (I.A.A.); ntentol@med.uoa.gr (N.T.); 3 University Research Institute of Maternal and Child Health and Precision Medicine and UNESCO Chair in Adolescent Health Care, National and Kapodistrian University of Athens, 11527 Athens, Greece; hkoniari@med.uoa.gr (E.K.); vefthymiou@outlook.com (V.E.); georgkon95@yahoo.gr (K.K.G.); chrousos@gmail.com (G.C.); 4 Department of Public and Community Health, School of Public Health, University of West Attica, 11521 Athens, Greece; pch23679083@uniwa.gr; 5 Infectious Diseases Unit, Pathophysiology Department, Medical School, National and Kapodistrian University of Athens, General Hospital of Athens Laiko, 11527 Athens, Greece; dimitris.bassoulis@gmail.com; 6 Department of Internal Medicine, General Hospital of Athens “ELPIS”, 11522 Athens, Greece; peggigrigo@yahoo.gr; 7 Department of Forensic Medicine and Toxicology, School of Medicine, National and Kapodistrian University of Athens, 11527 Athens, Greece

**Keywords:** COVID-19, SARS-CoV-2, coronavirus, telomeres, telomere length, severity

## Abstract

**Background**: Telomere attrition has been implicated in immune function and vulnerability to infectious diseases. However, the relation between telomere length and COVID-19 severity remains unclear. **Methods**: In this cross-sectional study, patients aged 30–75 years, with confirmed SARS-CoV-2 infection, as well as age- and BMI-matched controls without COVID-19, were recruited over a period of 1 year (2021–2022) from the outpatient clinics and wards of the General Hospitals “Laiko” and “Elpis” in Athens, Greece. Telomere length, expressed as a telomere to single-copy gene (T/S) ratio, was measured in all participants using a quantitative PCR-based method. Participants’ clinical, biochemical, demographic, and respiratory parameters were assessed in relation to their telomere length. **Results**: Study participants included a total of 139 individuals divided into three groups: controls (n = 34), patients with non-severe COVID-19 (n = 50), and patients with severe COVID-19 (n = 55). Patients with severe COVID-19 had significantly shorter telomeres when compared to both the non-severe COVID-19 group and controls (*p* < 0.001). Logistic regression analysis confirmed that telomere length was independently associated with disease severity (*p* < 0.001). Females demonstrated longer telomeres than males (*p* = 0.039), but no significant correlation was found between telomere length and age. When patients with non-severe and severe COVID-19 were analyzed together, no significant difference in telomere length was observed compared to controls (*p* = 0.727). **Conclusions**: Shortened telomeres may be linked to more severe forms of COVID-19, suggesting a potential role for telomere biology in disease progression. Results highlight the need for further research into telomere dynamics as a biomarker for disease susceptibility and outcome in viral infections.

## 1. Introduction

COVID-19, caused by the coronavirus SARS-CoV-2, first appeared in late 2019 and has subsequently affected millions of people globally [1]. Its clinical presentation varies significantly, ranging from mild or no symptoms to severe pneumonia, acute respiratory distress, and death [1]. Although significant advancements have been made in the understanding of COVID-19, the underlying factors contributing to the marked variability in individual responses remain only partially understood [1].

Telomeres are specialized DNA-protein structures located at the ends of chromosomes, essential for preserving genomic stability by protecting chromosomal integrity during cell division [2]. With each successive round of cell division, telomeric sequences progressively shorten, ultimately constraining the cell’s replicative capacity. This attrition is regarded as a hallmark of biological aging and has been associated with an increased risk of age-related diseases [2].

Emerging evidence indicates that infections may play a role in telomere attrition, primarily mediated by chronic inflammation and oxidative stress [3,4,5]. Persistent viral infections, including cytomegalovirus and herpes simplex virus-1, have been associated with shortened telomeres, potentially contributing to accelerated cellular aging and impaired immune competence [4,5]. Several studies have examined whether people with COVID-19 have different telomere lengths compared to those without COVID-19, but the results so far have not been consistent [6,7,8,9,10,11,12]. Similarly, research on how telomere length relates to the severity of illness has produced mixed outcomes [6,7,8,9,11,13,14,15,16,17,18,19,20]. These variations suggest that more focused research is needed to better understand how telomere biology might influence the course of COVID-19. The necessity for further investigation is not limited to SARS-CoV-2, as growing evidence suggests that telomere dynamics may also play a role in determining vulnerability to and progression of various other infectious diseases [4,5,21]. This underscores the potential utility of telomere length as a broader indicator of immune system robustness.

Given the uncertainty surrounding the relationship between telomere length and COVID-19, the present study aimed to assess potential associations between telomere length and the severity of SARS-CoV-2 infection and investigate whether telomere length correlates with patients’ demographic characteristics, medical history, respiratory support requirements, and biochemical parameters. By evaluating these parameters together, we sought to elucidate the broader clinical significance of telomere dynamics in the context of COVID-19.

## 2. Materials and Methods

### 2.1. Study Population

Study participants included patients, aged 30–75 years, with confirmed SARS-CoV-2 infection, as well as individuals without COVID-19, matched for age and BMI, who served as controls. The control group consisted of patients hospitalized or attended outpatient clinics for reasons unrelated to COVID-19. Participants were recruited over a period of 1 year (2021–2022) from the outpatient clinics and wards of the General Hospitals “Laiko” and “Elpis” in Athens, Greece. Participants diagnosed with COVID-19 were stratified into two groups according to the World Health Association (WHO) severity classification for COVID-19 [15]. The severe COVID-19 group included individuals with oxygen saturation below 90% on room air, clinical signs of pneumonia, or evidence of severe respiratory distress (e.g., use of accessory respiratory muscles, inability to complete full sentences, or a respiratory rate exceeding 30 breaths per minute in adults). The non-severe COVID-19 group comprised individuals who did not meet any of the criteria for severe disease.

The exclusion criteria encompassed individuals with a diagnosis of diabetes mellitus, those receiving immunosuppressive or immunomodulatory therapies, and individuals with malignancies, including hematological cancers. Moreover, participants with chronic kidney disease at stages 4–5 [estimated glomerular filtration rate (eGFR) < 30], severe liver disease or severe obesity [body mass index (BMI) > 40 kg/m^2^)] were excluded from the study.

The study protocol received approval from the Ethics Committee of the “Elpis” and “Laiko” General Hospitals (protocol 122/15-04-2021) and was conducted in accordance with the principles outlined in the Declaration of Helsinki. The study’s objectives were clearly communicated to all participants, and written informed consent was obtained prior to their enrollment.

### 2.2. Clinical and Laboratory Parameters

Upon enrollment in the study, the following data were recorded: demographic, personal, and family medical history; initial symptoms and findings from the clinical examination; and laboratory results obtained at hospital admission. Weight and height were measured in light clothing and body mass index (BMI, in kg/m^2^) was calculated. Participants were inquired about their medical history, including past or present diseases, medication usage, and smoking habits. They were classified as ex-smokers if they had ceased smoking for over two years, current smokers, or non-smokers. Arterial hypertension was defined if patients were on antihypertensive treatment. Coronary artery disease was defined as a history of angina, myocardial infarction, percutaneous transluminal coronary angioplasty or coronary artery bypass grafting. Cerebrovascular disease was defined as a history of stroke or revascularization at the carotid arteries. Chronic Obstructive Pulmonary Disease (COPD) was defined based on a documented history of the condition. A disabled person was defined as an individual with a physical, mental, intellectual, or sensory impairment that, in interaction with various barriers, hindered their full and effective participation in society on an equal basis with others.

Laboratory data included hematological markers (white blood cells, neutrophils, lymphocytes, monocytes, platelets, hemoglobin), biochemical markers and electrolytes [sodium, potassium, calcium, chloride, total bilirubin, alanine aminotransferase (ALT), aspartate aminotransferase (AST), gamma-glutamyl transferase (GGT), alkaline phosphatase (ALP), total protein, albumin, urea, creatinine, glucose, creatine kinase (CK), high sensitive troponin, lactate dehydrogenase (LDH)], inflammation markers [C-reactive protein (CRP), erythrocyte sedimentation rate, procalcitonin, serum ferritin], and coagulation markers (prothrombin time, activated partial thromboplastin time, fibrinogen, D-dimers). Any of the above parameters not routinely measured during hospitalization were analyzed in serum samples obtained specifically for the study.

Imaging, such as chest X-rays, was considered characteristic of COVID-19 in the presence of consolidation and ground-glass opacities, with a bilateral, peripheral distribution in the lower lung fields.

All patients were assessed up to the point of hospital discharge. Additionally, a follow-up communication took place one month after discharge to document the status of the individuals enrolled in the study.

### 2.3. Measurement of Telomeres

For all participants, a total of 2–4 cc of whole blood was drawn in EDTA tubes. Within 4 h, samples were transported for analysis to the Center for Adolescent Medicine and UNESCO Chair in Adolescent Health Care of the First Department of Pediatrics, Medical School, National and Kapodistrian University of Athens at the Aghia Sophia Children’s Hospital, and aliquoted into 2 tubes (Sardstedt SRS-72-694-006) of 1–2 mL each and frozen at −20 °C pending simultaneous DNA extraction and analysis. Genomic DNA was extracted using the Nucleospin Blood kit protocol [22]. LTL was then measured according to previously published protocols [12,23,24,25,26].

Telomere (“T”) and single copy gene (human albumin, “S”) lengths were measured via quantitative polymerase chain reaction (qPCR) using a Roche LC480 real-time PCR machine (Roche Diagnostics Corporation, Indianapolis, IN, USA). Samples were run in triplicate on 96-well assay plates in a Roche LightCycler 480 (Hoffmann-La Roche Ltd., Basel, Switzerland). Repeated measures of the T/S ratio in the same DNA sample gave the lowest variability when the sample well position for T PCR on the first plate matched its well position for S PCR on the second plate. When one sample’s duplicate T/S values differed by greater than 7%, the sample was run a third time and the two closest values were averaged to give the final result.

Relative telomere length determination by qPCR measures the ratio of telomere (T) signals, specific to the telomere hexamer repeat sequence TTAGGG, to autosomal single copy gene (S) signals. This ratio is normalized by control DNA samples to yield relative standardized T/S ratios proportional to average telomere length. In this technique reactions are performed independently, so a standard curve of pooled gDNA samples is utilized to assess the amount of each signal, while compensating for inter-plate variations in PCR efficiency. A standard curve [5 concentrations of pooled reference DNA samples prepared by serial dilution (100 to 6.25 ng/μL)] and randomly located internal QC sample replicates (n = 5), utilized as calibrator samples, to guide analysis and indicate overall quality of assay performance. Additionally, an NTC was added to random well locations to provide a unique fingerprint for each plate. All experimental and control samples were assayed in triplicate on each assay plate for both assays described previously [25].

The telomere (T) concentration was divided by the albumin (Alb) concentration (S) to yield a raw T/S ratio. The raw T/S ratio is divided by the average raw T/S ratio of the internal QC calibrator samples, within the same plate set, to yield a standardized T/S ratio to normalize results in reference to the same individual. Z-scores are calculated to adjust RTL in case differences in dynamic range are introduced by systematic differences between batches [25].

### 2.4. Statistical Analysis

All statistical analyses were conducted using statistical software IBM© SPSS© version 25 (IBM Statistical Package for Social Sciences for Windows, Version 25.0. Armonk, NY, USA: IBM Corp).

The variables are described using absolute, relative frequencies (%), mean ± standard deviation (SD) or median (interquartile range). The differences between groups investigated using the chi-square test, *t*-test for independent samples, Analysis of Variance (ANOVA) or Kruskal–Wallis H according to the data (qualitative, quantitative) and hypothesis of normality (using Kolmogorov–Smirnov, Shapiro–Wilk tests, skewness and kurtosis). Post hoc analysis for the 3 groups of the study was conducted using Tukey’s Honest Significant Difference test, Mann–Whitney U test or Games-Howell’s procedure dependent on homoscedasticity hypothesis. Logistic regression analysis was performed with COVID-19 severity as dependent variable and sex, age, BMI, smoking status, oxygen levels, and telomere length as independent variables. The statistically significant level was set at 0.05 two-sided.

## 3. Results

### 3.1. Demographic Characteristics of Study Participants

A total of 139 participants (105 patients and 34 controls) were enrolled in the study and were classified into three groups: control group (n = 34), non-severe COVID-19 group (n = 50), and severe COVID-19 group (n = 55). The proportion of female participants was significantly lower in the severe COVID-19 group compared to the control and non-severe groups. No significant differences were observed among the groups in terms of age, BMI, or smoking status (*p* = 0.134, *p* = 0.425, and *p* = 0.381, respectively). The demographic characteristics of the study participants are presented in Table 1.

### 3.2. Medical History and Respiratory Support of the Study Participants

Medical history and symptoms for all groups are presented in Table 2. The prevalence of hypertension, coronary heart disease, cerebrovascular disease, and disability did not significantly differ among the three groups. A borderline significant difference was observed in the use of statins, with a higher prevalence in the non-severe COVID-19 group compared to the control group (*p* = 0.014). Additionally, there was a trend toward a difference in the prevalence of COPD among the three groups (*p* = 0.050).

Regarding symptomatology and respiratory support, dyspnea was significantly more common in the severe COVID-19 group compared to the non-severe group (*p* < 0.001). Oxygen use did not significantly differ between the severe COVID-19 group and the control group. No significant difference was observed between the groups in the use of non-invasive mechanical ventilation [bilevel positive airway pressure (BiPAP), continuous positive airway pressure (CPAP)], high flow nasal cannula or the requirement for intubation and intensive care unit (ICU) admission.

### 3.3. Biochemical Characteristics of the Study Participants

The biochemical profiles of the study participants, as outlined in Table 3, revealed significant variations across the three groups. Hemoglobin levels did not differ significantly between groups (*p* = 0.939). However, platelet counts were significantly lower in the severe COVID-19 group than the non-severe group (*p* = 0.014). No significant differences were observed in white blood cell or neutrophil counts across groups. Lymphocyte counts were significantly higher in non-severe patients compared to both controls (*p* = 0.030) and severe cases (*p* = 0.007), suggesting lymphopenia associated with disease severity. Serum glucose levels showed significant differences (*p* = 0.001), being higher in severe COVID-19 cases than non-severe (*p* = 0.001) and control groups (*p* = 0.004). Serum sodium levels were also significantly lower in the severe group than the non-severe group (*p* = 0.024). No statistically significant differences were observed for potassium, total bilirubin, urea, or CK levels. CRP and ferritin levels were significantly elevated in severe cases. Similarly, lactate dehydrogenase (LDH) levels were significantly elevated in the severe group (*p* = 0.001), as were liver enzymes AST (*p* < 0.001), ALT (*p* < 0.001), and GGT (*p* = 0.005), reflecting hepatic involvement in severe disease. Albumin levels were significantly reduced in severe COVID-19 patients compared to both controls and the non-severe group (*p* < 0.001 and *p* = 0.006, respectively), consistent with a negative acute-phase response. Vitamin D levels did not differ significantly across groups (*p* = 0.883). Serum creatinine levels showed small but statistically significant differences (*p* = 0.049), with lower values in the non-severe group than controls (*p* = 0.015). No significant differences were found in urea, INR, or APTT values among the groups. Fibrinogen levels were significantly elevated in severe cases compared to controls (*p* = 0.016) and the non-severe group (*p* = 0.014). Notably, telomere length, expressed as the T/S ratio, was significantly reduced in severe COVID-19 cases, compared to both the control and non-severe groups (*p* < 0.001 for all comparisons), suggesting a potential link between telomere attrition and disease severity.

**Figure 1:**
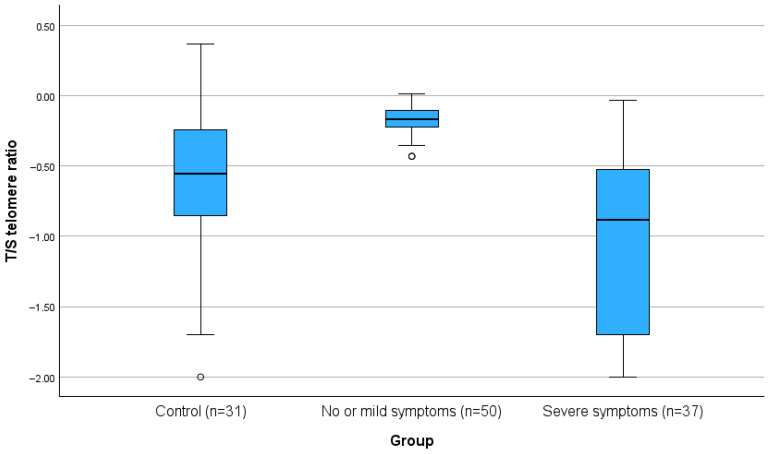
Boxplot of T/S telomere ratio in controls, non-severe, and severe COVID-19 patients (*p* < 0.001).

The T/S telomere ratio was significantly lower in people with severe COVID-19 compared to the non-severe group and the control group (all *p* < 0.001). For the primary outcome of the study, the telomeres, the differences are also presented in Figure 1.

Telomere length exhibited statistically significant differences between genders (independent of group interaction), with females demonstrating longer telomeres than males (*p* = 0.039). No significant correlation was observed between telomere length and age. Multiple regression analyses were performed with telomere length as the dependent variable and sex, age, BMI, smoking status, and oxygen use as independent variables. These analyses were conducted for the total sample as well as separately for each subgroup. No statistically significant models were identified in any of the analyses (*p* = 0.695, *p* = 0.538, *p* = 0.344, *p* = 0.501, respectively).

Additionally, logistic regression analysis was performed with COVID-19 severity (severe COVID-19 vs. non-severe COVID-19/controls) as dependent variable and sex, age, BMI, smoking status, oxygen levels, and telomere length as independent variables. Shorter telomere length was independently associated with COVID-19 severity (b = -4.39, *p* < 0.001, OR = 0.044, 95% CI = 0.007–0.262).

The T/S telomere ratio in the control group was 0.25 (0.10–0.57), compared to 0.45 (0.02–0.70) in individuals with non-severe and severe COVID-19 (groups 2 and 3 combined). This difference was not statistically significant (*p* = 0.727).

### 3.4. Quality Control

**Figure 2:**
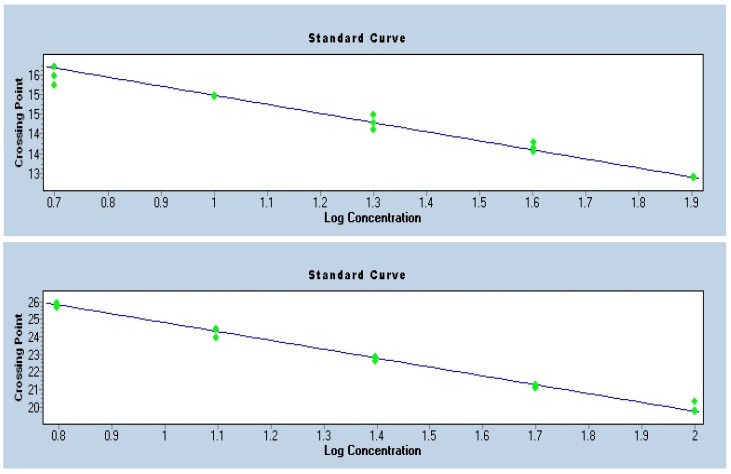
Standard Curves—Telomere and Albumin.

To ensure the accuracy of telomere length quantification by qPCR, standard curves were generated for the telomere repeat assay and for the single-copy reference gene albumin. Both standard curves exhibited strong linearity (R^2^ > 0.99) and slopes within the acceptable range, corresponding to amplification efficiencies close to 100% (Figure 2). The consistency of the Ct values across serial dilutions confirmed reliable assay performance and absence of major technical artifacts. These quality control measures validated the use of the telomere-to-single-copy gene ratio for the calculation of relative telomere length.

## 4. Discussion

This study found that patients with severe COVID-19 exhibited shorter telomere lengths when compared to both non-severe COVID-19 patients and controls. Logistic regression analysis further identified telomere length as an independent predictor of disease severity. Although females displayed longer telomeres than males, telomere length did not show a significant association with age. No significant difference in telomere length was observed between the overall COVID-19 cohort and controls.

Telomeres are specific DNA-protein complexes located at the terminal regions of chromosomes, playing a vital role in preserving genomic integrity and preventing chromosomal end-to-end fusions [2]. With each cell division, telomeres progressively shorten, leading to a decline in the replicative capacity of cells over time [2]. This progressive shortening has been strongly linked to aging, as it limits cellular proliferation and promotes senescence. Consequently, telomere length is considered a biological clock that helps determine the lifespan of individual cells and the organism as a whole [2].

Growing evidence suggests that infections may contribute to telomere shortening, primarily through mechanisms such as chronic inflammation and oxidative stress [3,4,5]. Studies have linked persistent viral infections, such as cytomegalovirus and herpes simplex virus-1, to reduced telomere length, which may accelerate cellular aging and compromise immune function [4,5]. However, the diversity in infection types, disease severity, and methods used to assess telomere length has led to inconsistencies across studies, making it difficult to establish definitive conclusions [3].

At the onset of the COVID-19 pandemic, we hypothesized that telomere length could play a role in disease severity [27]. This hypothesis was based on the observation that nearly all well-established risk factors for severe infection—such as male sex, obesity, diabetes, hypertension, coronary artery disease, chronic kidney disease, smoking, and COPD—are also linked to shortened telomeres [27]. The striking overlap between these conditions and telomere attrition suggested a potential connection between telomere biology and vulnerability to SARS-CoV-2 [27].

### 4.1. Telomere Length in Severe vs. Non-Severe COVID-19

Evidence regarding the association between infection severity and telomere length remains inconclusive, despite numerous studies investigating this relationship [6,7,8,9,11,13,14,15,16,17,18,19,20]. Several studies, have reported an association between telomere length and the severity of COVID-19 infection [7,9,13,14,15], while others have found no such link [11,16,17,18,19]. A 2023 meta-analysis, incorporating several of the aforementioned studies, sought to further elucidate this association [28]. A total of seven studies [7,9,14,15,19,20] comprising 1332 participants with severe COVID-19 and 6321 participants with non-severe COVID-19, were included in the meta-analysis [28]. The estimated pooled mean difference in leukocyte telomere length between severe and non-severe COVID-19 was 0.39 (95% CI: 0.02 to 0.81, I^2^ = 93.5%), indicating substantial heterogeneity [28]. According to the authors, these results failed to demonstrate a clear link between reduced telomere length and COVID-19 severity. In addition, three bidirectional mendelian randomization studies (not included in the meta-analysis) demonstrated that leucocyte telomere length was not causally related to severe COVID-19, nor was critical COVID-19 causally linked to telomere length [13,16,17,18].

A key limitation of these studies is that telomere length was measured after SARS-CoV-2 infection, making it difficult to determine whether telomere shortening preceded the infection or was a consequence of it. To address this issue, Wang et al. linked leucocyte telomere length values obtained from participants recruited into the UK Biobank between 2006 and 2010 with adverse COVID-19 outcomes recorded by 30 November 2020. They concluded that shorter leucocyte telomere length is associated with a higher risk of adverse COVID-19 outcomes, independent of several major risk factors for COVID-19, including age [15]. In another prospective study, telomere length was measured in 70 hospitalized COVID-19 patients and 491 healthy volunteers [13]. The study found a higher frequency of individuals with short telomeres among hospitalized COVID-19 patients, indicating that this group had significantly shorter telomeres compared to the reference population. Furthermore, short telomere length was independently associated with an increased risk of ICU admission and/or death, irrespective of age [13].

The novelty of our study lies in its severity-based approach, which spans the entire clinical spectrum of COVID-19 and thus provides greater granularity than simple case–control comparisons. By stratifying patients into non-severe and severe groups, we provide more granular evidence that shorter telomeres are specifically linked to severe forms of the disease, thereby complementing previous research and addressing a gap left by earlier studies. Taken together, these findings highlight the complexity of the relationship between telomere length and COVID-19 severity. Further large-scale, longitudinal studies, ideally with pre-infection telomere measurements, are needed to clarify the temporal and causal nature of this association.

### 4.2. Mechanisms Linking COVID-19 Infection and Telomere Length

Our findings indicate that patients with severe SARS-CoV-2 infection exhibit shorter telomeres compared to individuals with mild disease and uninfected controls. This phenomenon may be driven by multiple related biological processes. The key contributing factor is the intense inflammatory response triggered by COVID-19. Inflammation is known to have a profound impact on telomere length and telomerase activity across various major pathologies [29,30]; however, the specific molecular mechanisms underlying this association have yet to be fully elucidated [30]. Pro-inflammatory cytokines, especially interleukin-6 (IL-6) and tumor necrosis factor-alpha (TNF-α), have been closely linked to telomere shortening and reduced telomerase activity [30]. Elevated IL-6 and TNF-α enhance oxidative stress, resulting in increased oxidative DNA damage specifically targeting telomere regions, thus accelerating telomere shortening. Additionally, these cytokines can downregulate the activity and expression of telomerase reverse transcriptase (TERT), impairing cellular mechanisms responsible for telomere repair and preservation [30]. Consequently, persistent elevations of IL-6 and TNF-α—often seen in patients with severe COVID-19—may substantially contribute to accelerated cellular aging through both telomere erosion and diminished telomere maintenance [31]. In addition, the heightened replication of immune cells required to mount an effective response to viral infection increases cellular turnover, placing additional stress on telomeres and accelerating their attrition. Moreover, SARS-CoV-2 may compromise telomere integrity by disrupting telomere-maintenance mechanisms, including impaired telomerase activity and interference with protective telomere-associated proteins.

### 4.3. Association of Telomere Length with Measured Parameters

Interestingly, no significant association was observed between telomere length and age. The absence of a significant association between telomere length and age in our study may be attributable to the characteristics of the study population. The limited variability in participants’ ages may have hindered our ability to capture age-related differences in telomere dynamics. Moreover, the relatively small sample size could have reduced the statistical power needed to detect subtle associations. It is also important to consider that the presence of SARS-CoV-2 infection may have influenced telomere attrition through potentially overriding the typical pattern of age-related telomere shortening.

We also found that females had longer telomeres than males a difference that has been consistently observed across various studies and age groups. Women have longer telomeres than men, on average, due to a combination of genetic, hormonal, and developmental factors. This sex difference is evident from birth and persists throughout life. Estrogen exert a protective effect by enhancing telomerase activity, reducing oxidative stress, and supporting DNA repair, thereby promoting telomere maintenance [32,33,34,35]. In addition, the presence of two X chromosomes in females provides a genetic advantage, as the X chromosome harbors multiple immune-related genes, including those implicated in telomere regulation and immune defense [36]. This chromosomal complement may enhance the ability of females to mount stronger immune responses and mitigate telomere shortening, particularly in the context of systemic stressors such as SARS-CoV-2 infection [36]. These findings are in line with prior evidence suggesting that sex-related differences in telomere dynamics contribute to the generally greater resilience of women to age-related and infection-related cellular damage [36]. A meta-analysis from 36 cohorts (36,230 participants) showed that on average females had longer telomeres than males [33].

### 4.4. Telomere Length in COVID-19 Patients vs. Controls

Regarding telomere length between individuals with and without COVID-19, several observational studies have been published, however the findings have been inconsistent [6,7,8,9,10,11,12]. Benetos et al. provided some of the earliest published data in this field, demonstrating that telomere length is not associated with the presence of COVID-19 infection [6]. On the other hand, Kransiekov et al. reported that women who had recovered from COVID-19 exhibited significantly shorter telomeres compared to individuals in the control group [10]. It is important to note that their analysis was limited to female participants, whereas our cohort included both males and females. Given the well-established sex-related differences in telomere biology, this distinction may have contributed to the divergent findings between the two studies [10,32,33]. Similarly, Retuerto et al. demonstrated that hospitalized patients with COVID-19—94.4% of whom had pneumonia, indicating severe disease—exhibited significantly shorter baseline age-adjusted leukocyte telomere lengths compared to healthy controls [11]. The majority of these patients (94.4%) had pneumonia, indicating that the cohort predominantly comprised individuals with severe disease. Thus, their comparison was effectively between severe COVID-19 and uninfected controls, which aligns with our findings showing shorter telomere length specifically in patients with severe COVID-19, rather than across all infected individuals [11].

A recently published meta-analysis [37] which incorporated data from seven observational studies [6,7,8,9,10,11,12] aimed to clarify whether telomere length significantly differs between individuals with and without COVID-19. The meta-analysis included 1604 participants (967 COVID-19 patients and 690 uninfected controls). The pooled analysis demonstrated that COVID-19 patients had significantly shorter telomere length than non-infected individuals [SMD = −0.79, 95% confidence interval (CI): −1.16 to −0.17; *p* < 0.001]. There was substantial heterogeneity among studies (I^2^ = 91%) alongside a significant overall effect (Z = 2.62, *p* = 0.009) [9]. However, it is worth noting that some of the included studies had the methodological limitation of comparing only patients with severe COVID-19 to healthy controls, rather than examining the full clinical spectrum of disease severity. This may have amplified the observed effect size.

Further studies that include individuals across the full spectrum of COVID-19 severity -particularly non-severe cases—are needed to more accurately assess the relationship between telomere length and SARS-CoV-2 infection.

### 4.5. Limitations and Future Perspectives

This study has several limitations that should be acknowledged. Due to its cross-sectional design, we cannot establish whether shorter telomeres preceded SARS-CoV-2 infection or were a consequence of it. Telomere length was measured after diagnosis, which may reflect both pre-existing differences and changes related to the illness itself. Although we included a range of participants, the overall sample size was relatively modest, which may have limited the statistical power to detect more subtle associations. This is particularly relevant for subgroup analyses (e.g., by gender or other clinical characteristics), as smaller numbers within these groups reduce the reliability of such comparisons. In addition, the restricted age distribution may have further constrained our ability to identify age-related effects. Recruitment of a larger cohort was particularly challenging due to the constraints imposed by the COVID-19 pandemic. Furthermore, telomerase activity, which would have provided complementary information on the dynamic regulation of telomere biology, was not measured in our study and should be considered in future research.

Further investigation is essential to shed light on the role telomeres may play in shaping the body’s response to infections like COVID-19. Studies that follow individuals over time could help determine whether shortened telomeres are a predisposing factor for severe illness or a result of the infection itself. The observed link between infections and telomere biology points to a broader need for research into how these structures influence immune system behavior and overall disease risk. A deeper understanding in this area might open new pathways for identifying at-risk individuals and guiding future treatment approaches.

## 5. Conclusions

Our findings suggest that shorter telomeres are associated with increased severity of COVID-19, supporting the idea that telomere length may serve as a marker of vulnerability in the context of infectious diseases. Beyond COVID-19, telomere length holds potential clinical utility as a biomarker to identify individuals at higher risk for severe outcomes across different infectious conditions. These results contribute to the growing body of evidence linking cellular aging with disease progression and emphasize the need for longitudinal studies with pre-infection telomere measurements to establish causality. Such studies could clarify whether telomere shortening is merely a correlate or an active contributor to poor outcomes, thereby informing future strategies for patient risk stratification and personalized interventions.

## Figures and Tables

**Table 1:** Demographic characteristics of the study participants.

	Total (n = 139)	Control (n = 34) Group 1	Non-Severe COVID-19 (n = 50) Group 2	Severe COVID-19 (n = 55) Group 3	*p* for All Groups	*p* 2 vs. 3	*p* 1 vs. 2	*p* 1 vs. 3
Female gender (n, %)	67 (48.2%)	22 (64.7%)	27 (54.0%)	18 (32.7%)	**0.008** ^†^	**0.028** ^†^	0.329 ^†^	**0.003** ^†^
Age (years)	53.52 ± 10.13	50.71 ± 9.57	53.66 ± 10.20	55.13 ± 10.21	0.134 ^‡^	-	-	-
BMI (kg/m^2^)	28.00 ± 4.83	28.38 ± 5.60	27.26 ± 4.29	28.43 ± 4.78	0.425 ^‡^	-	-	-
Smoking status (n, %)								
No	65 (53.7%)	13 (40.6%)	23 (56.1%)	29 (60.4%)	0.381 ^†^	-	-	
Ex smoker	31 (25.6%)	12 (37.5%)	10 (24.4%)	9 (18.8%)				
Current	25 (20.7%)	7 (21.9%)	8 (19.5%)	10 (20.8%)				

Data are presented as n (%) or mean ± SD (standard deviation). *p*-value is computed using ^†^ χ^2^ test or ^‡^ *t*-test for independent samples. Bold values indicate statistically significant differences.

**Table 2:** Medical history and respiratory support of study participants.

	Total (n = 139)	Control (n = 34) Group 1	Non-Severe COVID-19 (n = 50) Group 2	Severe COVID-19 (n = 55) Group 3	*p* for All Groups	*p* 2 vs. 3	*p* 1 vs. 2	*p* 1 vs. 3
Hypertension (n, %)	31 (22.3%)	7 (20.6%)	12 (24.0%)	12 (21.8%)	0.929 ^†^	-	-	-
Disabled (n, %)	21 (15.1%)	3 (8.8%)	9 (18.0%)	9 (16.4%)	0.487 ^†^	-	-	-
Coronary Heart Disease (n, %)	9 (6.5%)	2 (5.9%)	4 (8.0%)	3 (5.5%)	0.520 ^‡^	-	-	-
Cerebrovascular disease (n, %)	2 (1.4%)	0 (0.0%)	0 (0.0%)	2 (3.6%)	0.154 ^‡^	-	-	-
Reperfusion (n, %)	7 (5.0%)	3 (8.8%)	2 (4.0%)	2 (3.6%)	0.215 ^‡^	-	-	-
Statin (n, %)	20 (14.4%)	1 (2.9%)	11 (22.0%)	8 (14.5%)	0.051 ^†^	0.923 ^†^	**0.014** ^†^	0.078 ^†^
Chronic Obstructive Pulmonary Disease (n, %)	5 (3.6%)	0 (0.0%)	1 (2.0%)	4 (7.3%)	**0.050 ^‡^**	0.205 ^†^	0.407 ^†^	0.108 ^†^
Fever (n, %)	88 (63.3%)	26 (76.4%)	21 (42%)	41 (74.5%)	0.457 ^‡^	-	-	-
Cough (n, %)	42 (30.2%)	15 (44.1%)	6 (12%)	21 (38.2%)	0.181 ^†^	-	-	-
Dyspnea (n, %)	26 (18.7%)	8 (23.5%)	2 (4%)	16 (29.1%)	**0.003**	**<0.001 ^†^**	0.007 ^†^	0.566 ^†^
Anosmia (n, %)	3 (2.2%)	1 (2.9%)	2 (4%)	0 (0.0%)	0.267 ^‡^	-	-	-
Hardship (n, %)	28 (20.1%)	6 (17.6%)	8 (16%)	14 (25.5%)	0.443 ^†^	-	-	-
Diarrhea (n, %)	7 (5.0%)	4 (11.8%)	1 (2%)	2 (3.6%)	0.120 ^‡^	-	-	-
Oxygen use (n, %)	48 (34.5)	17 (50.0%)	0	31 (56.3%)	**<0.001** ^†^	**<0.001** ^†^	**<0.001** ^†^	0.558 ^†^
Nasal Oxygen (n, %)	23 (16.5)	9 (26.5%)	0	14 (25.5%)	**<0.001** ^†^	**<0.001** ^†^	**<0.001** ^†^	0.915 ^†^
Venturi Mask (n, %)	32 (23.0)	11 (32.4%)	0	21 (38.2%)	**<0.001** ^†^	**<0.001** ^†^	**<0.001** ^†^	0.578 ^†^
BiPAP/CPAP (n, %)	1 (0.7)	1 (2.9%)	0	0 (0.0%)	0.211 ^†^	-	-	-
High flow nasal cannula (n, %)	5 (3.6)	3 (8.8%)	0	2 (3.6%)	0.103 ^†^	-	-	-
Intubation /ICU (n, %)	2 (1.4)	0 (0.0%)	0	2 (3.6%)	0.212 ^†^	-	-	-

Data are presented as n (%), mean ± SD (standard deviation), median value (25, 75 percentile). *p*-value is computed using ^†^ χ^2^ test or ^‡^ Monte Carlo simulation method. Bold values indicate statistically significant differences. BiPAP: bilevel positive airway pressure, CPAP: continuous positive airway pressure, ICU: intensive care unit.

**Table 3:** Biochemical characteristics of study participants.

	Total (n = 139)	Control (n = 34) Group 1	Non-Severe COVID-19 (n = 50) Group 2	Severe COVID-19 (n = 55) Group 3	*p* for All Groups	*p* 2 vs. 3	*p* 1 vs. 2	*p* 1 vs. 3
Hemoglobin (mg/dL)	13.7 ± 1.6	13.8 ± 1.9	13.7 ± 1.6	13.7 ± 1.5	0.939	-	-	-
Platelets/μL	212,000 (158,000, 272,000)	212,000 (150,250, 268,250)	253,000 (185,000, 282,000)	194,000 (146,000, 259,000)	**0.014** ^‡^	**0.014** ^†^	0.132 ^†^	0.685 ^†^
White blood cell/μL	6040 (4520, 7705)	5200 (4345, 6865)	6510 (5045, 8005)	5600 (4200, 8210)	0.259 ^‡^	-	-	-
Neutrophils/μL	3700 (2350, 5390)	3200 (2275, 4525)	3900 (3000, 5200)	3850 (2175, 6215)	0.971 ^‡^	-	-	-
Lymphocytes /μL	1346.0 ± 865.6	1213.7 ± 760.5	1627.7 ± 926.9	1173.8 ± 818.3	**0.016**	**0.007**	**0.030**	0.830
Vitamin D (ng/mL)	21.0 (14.0, 29.0)	21.0 (9.8, 25.5)	20.0 (16.3, 28.8)	23.0 (13.0, 29.0)	0.883 ^‡^	-	-	-
Glucose (mg/dL)	105.0 (94, 123)	109.5 (95.8, 126.5)	97.5 (88.8, 106.3)	110.0 (97.0, 128.0)	**0.001** ^‡^	**0.001** ^†^	**0.004** ^†^	0.953 ^†^
Sodium (mmol/L)	138.8 ± 3.1	138.3 ± 3.9	139.6 ± 2.2	138.3 ± 3.1	**0.040**	**0.024** ^†^	**0.039** ^†^	0.949 ^†^
Potassium^‡^ (mmol/L)	4.3 (3.9, 4.6)	4.2 (3.8, 4.7)	4.4 (4.1, 4.6)	4.3 (3.9, 4.6)	0.309	-	-	-
Hs Trop (ng/L)	5.0 (3.0, 9.0)	8.0 (4.3, 11.5)	5.0 (3.0, 6.0)	6.0 (3.0, 8.3)	0.298 ^‡^	-	-	-
Total bilirubin (mg/dL)	0.4 (0.3, 0.6)	0.4 (0.3, 0.6)	0.4 (0.3, 0.7)	0.4 (0.3, 0.6)	0.337 ^‡^	-	-	-
Albumin (g/L)	41.0 (30.7, 45.0)	40.00 (29.5, 43.0)	44.5 (41.4, 46.0)	37.5 (33.6, 42.8)	**<0.001** ^‡^	**<0.001** ^†^	**0.006** ^†^	0.342 ^†^
CK (mcg/L)	105.0 (61.5, 191.5)	119.0 (70.0, 219.5)	105.0 (60.0, 202.0)	91.0 (54.50, 172.5)	0.442 ^‡^	-	-	-
Urea ^‡^ (mg/dL)	33.0 (25.0, 39.3)	33.5 (27.3, 39.5)	33.0 (25.8, 39.3)	33.0 (24.8, 40.8)	0.770 ^‡^	-	-	-
Creatinine ^‡^ (mg/dL)	0.8 (0.7, 1.0)	0.9 (0.7, 1.1)	0.8 (0.6, 0.9)	0.8 (0.7, 1.1)	**0.049** ^‡^	**0.049** ^†^	**0.015** ^†^	0.557 ^†^
CRP ^‡^ (mg/dL)	6.1 (1.8, 24.5)	10.6 (1.9, 33.4)	3.0 (1.4, 10.0)	8.3 (2.4, 45.4)	**0.025** ^‡^	**0.025** ^†^	**0.029** ^†^	0.913 ^†^
Ferritin (ng/mL)	300.0 (106.0, 546.0)	365.0 (243.0, 715.0)	150.5 (62.3, 252.8)	359.5 (139.3, 809.5)	**<0.001** ^‡^	**<0.001** ^†^	**<0.001** ^†^	0.978 ^†^
LDH (U/L)	242.5 (193.3, 332.8)	262.0 (207.0, 368.5)	205.0 (180.0, 244.5)	291.0 (203.0, 343.5)	**0.001** ^‡^	**0.001** ^†^	**0.002** ^†^	0.885 ^†^
AST (U/L)	28.0 (20.0, 42.5)	32.5 (22.5, 45.0)	23.0 (17.0, 30.3)	37.0 (22.5, 58.5)	**<0.001** ^‡^	**<0.001** ^†^	**<0.001** ^†^	0.309 ^†^
ALT (U/L)	29.0 (18.5, 43.0)	30.0 (23.0, 36.3)	23.5 (16.0, 31.0)	38.0 (22.5, 59.0)	**<0.001** ^‡^	**0.001** ^†^	**0.043** ^†^	0.071 ^†^
GGT (U/L)	31.0 (19.5, 51.5)	38.0 (28.5, 55.3)	23.0 (14.5, 37.0)	36.5 (21.8, 60.3)	**0.005** ^‡^	**0.005** ^†^	**0.004** ^†^	0.741 ^†^
ALP (IU/L)	64.5 (52.0, 85.0)	60.50 (51.0, 73.3)	65.0 (52.0, 85.0)	68.0 (52.5, 86.5)	0.673 ^‡^	-	-	-
Fibrinogen (mg/dL)	506.8 ± 159.6	446.2 ± 75.5	445.4 ± 196.1	579.4 ± 150.2	**0.015**	**0.016**	0.990	**0.014**
INR	0.99 (0.95, 1.03)	0.98 (0.94, 1.07)	0.99 (0.96, 1.03)	0.99 (0.94, 1.02)	0.521 ^‡^	-	-	-
APTT	35.4 (33.5, 37.3)	35.2 (34.0, 38.6)	34.6 (31.6, 36.7)	35.6 (33.9, 37.8)	0.403 ^‡^	-	-	-
RATIO T/S TELOMERES	0.37 (0.02, 0.69)	0.25 (0.10, 0.57)	0.68 (0.59, 0.78)	0.02 (0, 0.23)	**<0.001** ^‡^	**<0.001** ^†^	**<0.001** ^†^	**<0.001** ^†^

Data are presented as mean ± SD (standard deviation), median value (25, 75 percentile). *p*-value is computed using Analysis of Variance (ANOVA), ^‡^ Kruskal–Wallis H or ^†^ Mann–Whitney U test. Bold values indicate statistically significant differences. Hs Trop: high-sensitivity troponin, CK: creatine kinase, CRP: C-reactive protein, LDH: lactate dehydrogenase, AST: aspartate aminotransferase, ALT: alanine aminotransferase, GGT: gamma-glutamyl transferase, ALP: alkaline phosphatase, INR: international normalized ratio, PT: prothrombin time, APTT: activated partial thromboplastin time.

## Data Availability

Data are available from the corresponding author upon reasonable request due to privacy and ethical restrictions.
